# Unilateral Anomalous Profunda Femoris Artery: An Anatomical Variation of Clinical Significance

**DOI:** 10.7759/cureus.32188

**Published:** 2022-12-04

**Authors:** Ariyanachi K, Rohini Motwani, Mrudula Chandrupatla

**Affiliations:** 1 Anatomy, All India Institute of Medical Sciences Bibinagar, Hyderabad, IND

**Keywords:** variation, profunda femoris artery, femoral triangle, femoral artery, anomalous

## Abstract

The profunda femoris artery (PFA) is the largest branch of the femoral artery (FA) in the femoral triangle and is the chief arterial supply for the adductors, flexors, and extensors of the thigh, as well as the hip joint and the femur. Unilateral anomalous origin and variant course of the PFA were observed during the routine cadaveric dissection for undergraduate teaching. Of note, the FA gave only a few muscular branches, whereas all the other significant arteries of the front of the thigh took their origin from the anomalous profunda femoris artery. Both interventional radiologists and surgeons must be familiar with the anatomical variants of the PFA and FA. The likelihood of many sources supplying the skin and pedicle, particularly in reconstructive surgery, requires surgeons to be cognizant of this subject hence crucial in order to minimize surgical complications.

## Introduction

The femoral artery (FA) and profunda femoris artery (PFA) manifest themselves in a vast range of patterns, many of which are closely connected to one another. The PFA arises from its lateral aspect approximately 3.5 cm distal to the mid-inguinal point. It then passes behind the FA and femoral vein on the pectineus and leaves the Scarpa triangle (femoral triangle) by passing through the interval between the pectineus and adductor longus and then descends in the gap between adductor longus and magnus and it terminates as the fourth perforator. It then pierces the adductor magnus and unites with the popliteal artery's branches to the muscles [1]. PFA sends out arteriae circumflexae femoris medialis (medial circumflex femoral artery [MCFA]), and arteria circumflexa femoris lateralis (lateral circumflex femoral artery [LCFA]) in the proximal part of the thigh, whereas some unnamed muscular branches and three perforating branches distally. PFA supplies the adductor, extensor, and flexor muscles of the thigh, and also anastomoses with the iliac (internal and external) arteries above and with the popliteal artery below. In addition, it also supplies the hip joint and the femur [2].

Because of their clinical implications, anatomical variants of the PFA and its branches are of great interest to anatomists, radiologists, and surgeons [3]. Through collateral pathways in the lower pelvis, starting from the internal iliac artery, the PFA performs a vital compensatory function for auxiliary circulation in vascular occlusive disorders. If both aortoiliac and femoropopliteal lesions are present, the significance of this collateral pathway increases [4]. Understanding the precise placement of LCFA is critical when procuring an anterolateral thigh flap for corrective surgery, aorto-popliteal bypass, and extra/intracranial bypass surgeries [5]. It becomes crucial to understand the path of MCFA particularly in surgeries such as osteotomies (trochanteric and intertrochanteric) and total arthroplasty, as well as in interventional radiology during FA puncture, to avoid causing iatrogenic avascular necrotic lesions of the femoral head [6]. In situations of breast cancer, the branches of PFA are also utilized as a lengthy arterial stem for reconstructive surgery following mastectomy [7]. A unique pattern of PFA has been documented in this case report.

## Case presentation

**Figure 1:**
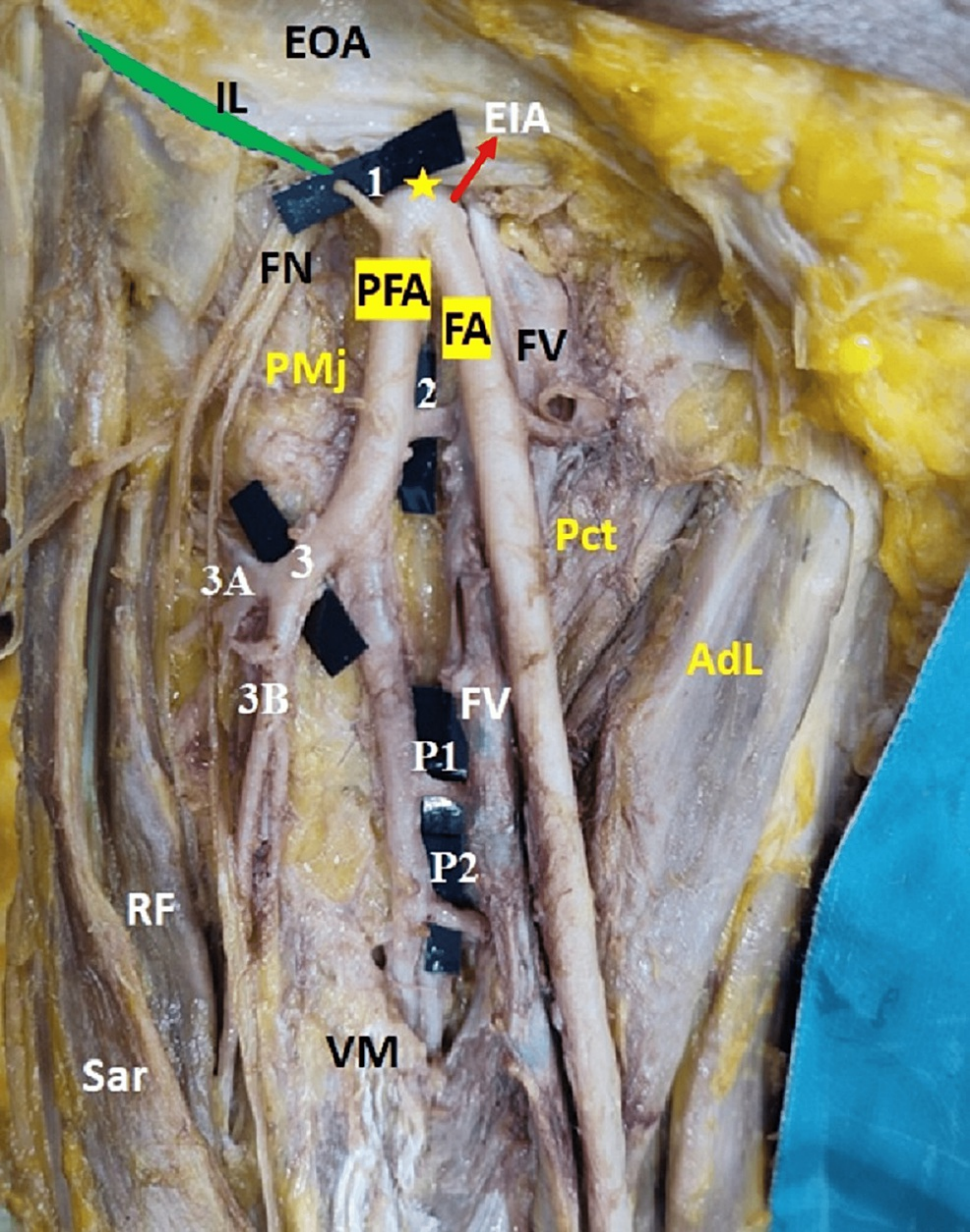
Showing anomalous bifurcation of External Iliac Artery (EIA) Anomalous bifurcation of EIA at the midinguinal point (yellow asterisk) with variant profunda femoris artery (PFA) of right femoral triangle. 1: Superficial epigastric artery, 2: Medial circumflex femoral artery (MCFA), 3: Lateral circumflex femoral artery (LCFA), 3A: Superior branch of LCFA (showing trifurcation into ascending, transverse and descending branches), 3B: Inferior branch of LCFA, P1: Perforator 1, P2: Perforator 2, FA: Femoral artery, IL: Inguinal ligament, FN: Femoral nerve, FV: Femoral vein, EIA: external iliac artery, EOA: External Oblique Aponeurosis, RF: Rectus femoris, Sar: sartorius, VM: Vastus medialis, PMj: Psoas major, Pct: Pectineus, AdL: adductor longus

During routine dissection of the lower limb for undergraduate medical students, we encountered a unique pattern of PFA in the right femoral triangle of a 68-year-old female cadaver. The external iliac artery (EIA) instead of continuing as FA, bifurcated at the midinguinal point into medial and lateral branches (Figure 1).

The medial branch (25 cm long; 1.59 mm wide) descended in the femoral triangle as FA, continued its course into the adductor canal, and entered the popliteal fossa as the popliteal artery via the adductor hiatus. It gave only muscular branches in the femoral triangle.

The lateral branch (12 cm long; 1.6 mm wide) which was on the psoas major muscle continued as PFA. It descended lateral to the femoral vein in the femoral triangle. It eventually exited the Scarpa triangle by traveling through the space between the vastus medialis and the adductor longus and terminated as the third perforator. The superficial epigastric artery arose from the lateral margin of the PFA, approximately 0.5 cm below the inguinal ligament. Around 3 cm well below the inguinal ligament, MCFA emerged from the medial border of PFA. Between the pectineus and psoas major, MCFA is then divided into an ascending and a transverse branch. The LCFA branched off from the lateral side of the PFA, approximately 5 cm below the inguinal ligament. It ran laterally beneath the rectus femoris, between the two divisions of the femoral nerve (FN), and split into superior and inferior branches. The superior branch is split into three branches: ascending, transverse, and descending. The inferior branch supplied the quadriceps femoris. The first perforator emerged from the medial aspect of the PFA approximately 7 cm well below the inguinal ligament, and the second perforator emerged from the medial aspect of the PFA approximately 9 cm below the inguinal ligament. The first and the second perforators ran deep to the adductor longus, below the adductor brevis, and anastomose with the terminal part of the PFA (Figure 1). The deep external pudendal artery, superficial circumflex iliac artery, and superficial external pudendal artery branches were absent on the right side. The diameter of the FA, PFA, and its branches were calibrated in millimeters using a digital vernier caliper. The diameter of both arteries was approximately the same. The left-sided FA and PFA showed normal branching patterns. There were no additional obvious anomalies or signs of surgeries in the femoral triangle of both side. Table 1 summarizes the key observations from our case.

**Table 1:** Showing summary of finding encountered in the present case report (Figures 1, 2).

Artery of right femoral triangle	Observations
External iliac artery (EIA)	Bifurcated at the mid-inguinal point : Medial and lateral branch
Femoral artery (FA)	As medial branch from EIA. Gave only 2-3 muscular branches. Continued as popliteal artery
Profunda femoris artery (PFA)	As lateral branch from EIA. Gave a small Medial and a large Lateral circumflex femoral artery (MCFA & LCFA) and two perforators. Continued as third perforator

## Discussion

The profunda femoris artery (PFA), also known as the deep artery of the thigh, normally arises from the lateral side of the FA, 3.75 cm distal to the mid-inguinal point [2]. In the present case, we encountered unilateral (right side) bifurcation of EIA at the midinguinal point, instead of continuing as FA (Figure 1). Although a few researchers have identified unilateral or bilateral extraordinary high origins of PFA in case reports and studies, the splitting of EIA into FA and PFA is uncommon [8]. In their study of two cadavers, Jadhav and colleagues noted a similar unilateral bifurcation of the FA on the left side (in both cases), i.e. PFA was emerging from the lateral aspect of the FA immediately behind the inguinal ligament [9]. Some authors observed that if the PFA originated near the mid-point of the inguinal ligament, the origin was then from the lateral aspect of FA [10]. An anatomical understanding of the level of origin of PFA is essential in order to prevent iatrogenic femoral arteriovenous fistulas caused by femoral artery puncture. Pseudo-aneurysm can be caused by percutaneous FA cannulation injury. When the puncture occurs at a PFA or FA distal to the PFA origin, this occurs more frequently. When employed in plastic and reconstructive surgery, understanding the origins of PFA is critical for avoiding flap necrosis, particularly in the tensor fascia latae [10]. It's also important in femoral hernia surgery and vascular reconstruction treatments in the proximal leg as the muscular branches are used in conjunction with myocutaneous flaps by plastic surgeons [11]. To better understand the general architecture of the femoral vessels and to aid in the staging of the catheterization, upper thigh ultrasonography, and evaluation can be done prior to undertaking upper thigh surgical operations and femoral vascular catheterization.

In the present case report, the PFA follows a unique pattern, resting parallel and lateral to the FA throughout its journey. It normally travels laterally at first, then spirals posterior to the femoral vein and FA to arrive at the medial aspect of the femur [2]. We could not find a similar variant course of PFA even an extensive literature review. With femoral triangle operations, variations with the PFA pattern and its branches are therapeutically necessary because they reduce the risk of intraoperative bleeding and postoperative complications [11]. In order to carry out intertrochanteric osteotomies, in addition to preventing iatrogenic vascular necrotic damage to the femoral head in hip reconstructive surgeries and posterior route fracture fixation of the acetabular lesion, Gautier et al. claim that a clear understanding of the anatomical variation of MCFA is necessary [12]. In the present report, CFA originated from PFA, traveled behind the two divisions of the FN, and separated into superior and inferior divisions, which differs from the typical branching pattern. Goel et al. discovered a similar unusual route of the LCFA posterior to the posterior division of the FN, as well as a thick branch to the vastus lateralis muscle [13].

Because these are widely used in extracranial-intracranial bypass, aortopopliteal bypass, and anterolateral thigh flaps for reconstruction, plastic surgeons should be aware of the diverse branching pattern of the LCFA [14]. When elevating free rectus femoris muscular flaps with branches of the posterior division of the FN for one-stage correction of facial paralysis, the surgeon should pay close attention to the unusual path of LCFA [15]. The arteriographic examination is also required for preoperative anatomical evaluation of LCFA. The use of the LCFA as a potential arterial graft for coronary artery bypass grafting is increasingly investigated. An aberrant course of LCFA during such surgical procedures may have the unintended consequence of causing harm to the branches of the FN traveling in front of LCFA [15]. An FN block is generally provided immediately above the origin of PFA in knee replacement surgeries. The absence of LCFA behind the posterior division of the FN may be misunderstood due to a lack of awareness of the presence of LCFA behind the latter, resulting in LCFA injury. Exploration of the FN for anesthetic treatments, then, necessitates knowledge of the LCFA's varied path with respect to the FN [13]. When employing sharp ending version guidewires during hip fracture surgery, anatomical understanding of LCFA branches is indeed important. Because of its unique path as in this case behind the FN, such surgical techniques involving the investigation of LCFA branches may result in iatrogenic damage to the ascending branch of LCFA [16]. In the present case, a superficial epigastric artery arose from the lateral side of PFA about 0.5 cm below the inguinal ligament. In a study done by Rajani et al., in 6.06% of the specimens, PFA gave origin to superficial branches [11]. Studies have reported that the probabilities of superficial branches from the FA are 83.3%, 6.6% from the deep circumflex artery, 6.6% from the LCFA, and 3.3% from the PFA [17].

The perforating arteries perforate the femoral attachment of the adductor magnus to reach the flexor compartment of the thigh. As such there are usually three perforating branches, and the fourth perforator artery is the continuation of the PFA [18]. In the present case, we reported the presence of three perforators (Figure 1) instead of the usual four. Two arose from PFA and the third one was its continuation. In the field of cosmetic and reconstructive surgery, getting suitable and visually pleasing skin and soft tissue cover for large, superficial tissue lesions has long been a difficulty, making up a sizeable portion of the plastic surgeon's effort. The flap's blood supply is crucial to its capacity to survive after being transplanted from one area of the body to another. For the perforator-based flaps, the lower limb is the largest donor site in the body [18]. Hence deep anatomical understanding of the perforators is essential for plastic surgeons.

**Figure 2:**
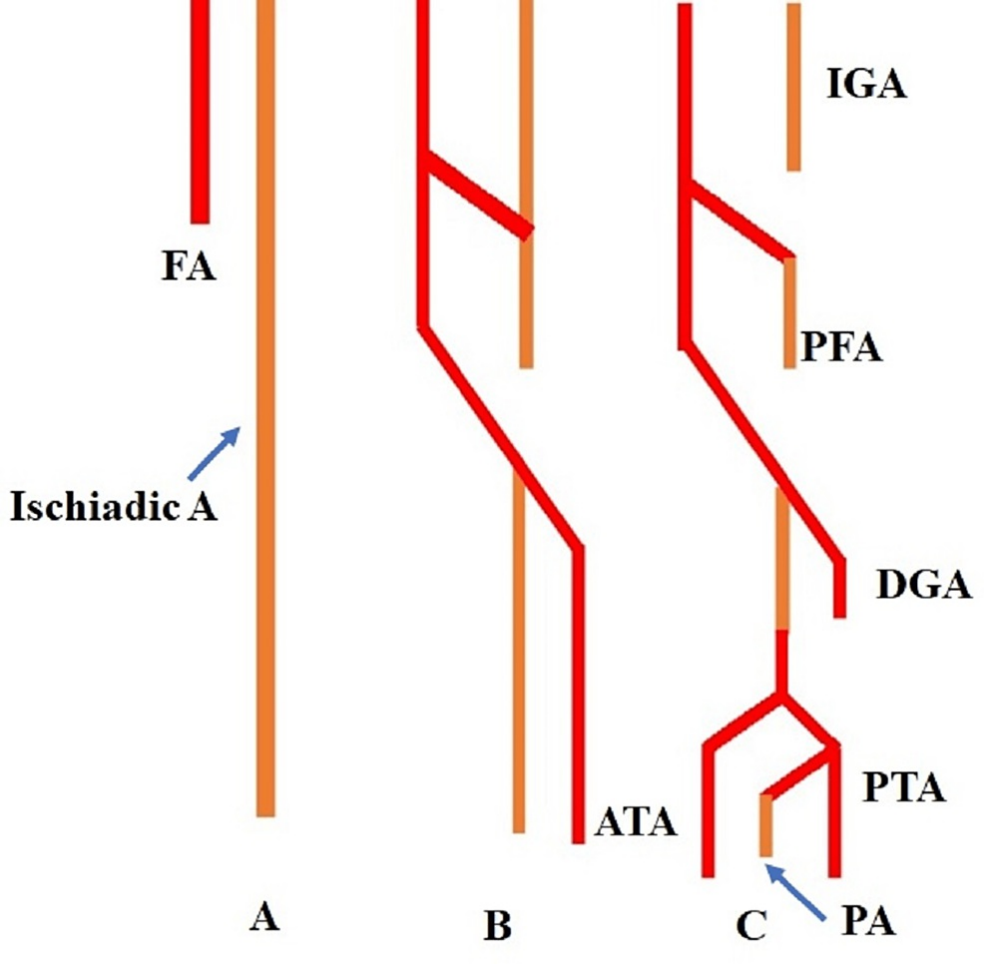
Schematic representation of the development of arteries of lower limb. Showing the development of arteries of lower limb. FA: Femoral artery, PFA: Profunda femoris artery, IGA: Inferior gluteal artery, DGA: Descending genicular A, ATA: Anterior tibial artery, PTA: Posterior tibial artery, PA: Peroneal artery.

The lower limb arteries branch from the fifth lumbar artery as a complex anastomotic network linking its two branches, the ventral femoral and the dorsal sciatic arteries, ultimately giving the PFA. Such variations may occur as a result of any deviation from the expected pattern of development (Figure 2) [19]. Some of the channels regress while others enlarge and produce a distinct vascular pattern during development. The communication that was meant to disappear persisted, resulting in various anomalies [20]. Because morphological and molecular alterations in the limb mesenchyme precede vascular development in the lower limb, differences in the vascular pattern are frequently seen. Precise embryological and anatomical knowledge of normal and variant PFA anatomy is therefore essential for surgeons, clinicians, and radiologists to prevent injury to the vessels in the Scarpa triangle while doing any procedures.

## Conclusions

In this report, we are presenting a rare case of unilateral bifurcation of the EIA with an anomalous course and branching of PFA. As FA and PFA are used in a wide range of clinical procedures, including embalming, doppler ultrasound imaging, arteriography, hemodialysis, reconstructive surgeries, and angioplasty. Surgeons, radiologists, and anatomists must be familiar with the various patterns of the vessels in the femoral triangle. Hence, we suggest high-resolution ultrasonography should be done prior to any intervention on vessels of the femoral region, which can provide anatomical and functional information about it, which would be helpful in planning various procedures.
